# Changes in MCP‐1, HGF, and IGF‐1 expression in endometrial stromal cells, PBMCs, and PFMCs of endometriotic women following 1,25(OH)2D3 treatment

**DOI:** 10.1111/jcmm.17592

**Published:** 2022-10-19

**Authors:** Sahel Heidari, Roya Kolahdouz‐Mohammadi, Sepideh Khodaverdi, Tahereh Mohammadi, Ali‐Akbar Delbandi

**Affiliations:** ^1^ Immunology Research Center, Institute of Immunology and Infectious Diseases Iran University of Medical Sciences Tehran Iran; ^2^ Department of Immunology, School of Medicine Iran University of Medical Sciences Tehran Iran; ^3^ Department of Nutrition, School of Public Health Iran University of Medical Sciences Tehran Iran; ^4^ Endometriosis Research Center Iran University of Medical Sciences Tehran Iran

**Keywords:** 1,25(OH)2D3, endometrial stromal cells, endometriosis, HGF, IGF‐1, MCP‐1, mononuclear cells

## Abstract

1,25(OH)2D3 has anti‐inflammatory and growth inhibitory effects. Our study explored the effect of 1,25(OH)2D3 treatment on the expression of monocyte chemotactic protein‐1 (MCP‐1), hepatocyte growth factor (HGF), and insulin‐like growth factor‐1 (IGF‐1) by peripheral blood mononuclear cells (PBMCs), peritoneal fluid mononuclear cells (PFMCs), endometrial stromal cells (ESCs), and its effect on the proliferation of PBMCs and PFMCs of patients with endometriosis compared with controls. PBMCs, PFMCs, and ESCs were obtained from 10 endometriosis patients and 10 non‐endometriotic individuals. After treating cells with 0.1 μM of 1,25(OH)2D3 for 6, 24, and 48 h, the gene and protein expression of mentioned factors were evaluated by real‐time PCR and ELISA methods, respectively. 1,25(OH)2D3 treatment significantly reduced the protein expression of MCP‐1, HGF, and IGF‐1 in PBMCs and PFMCs of endometriotic patients at 48 h (*p* < 0.05–<0.01). Also, this treatment significantly reduced *MCP‐1*, *HGF*, and *IGF‐1* gene and/or protein expression in EESCs and EuESCs at 24 and 48 h (*p* < 0.05–<0.01). 1,25(OH)2D3 treatment also reduced the proliferation of PBMCs and PFMCs of endometriotic patients compared with controls (*p* < 0.01). 1,25(OH)2D3 can be considered as a potentially effective agent in the prevention and treatment of endometriosis along with other therapies.

## 1 INTRODUCTION

Endometriosis as an enigmatic and often debilitating condition, described as the presence of endometrial‐like tissues in the uterine cavity.^1^ Endometriosis is the most common cause of chronic pelvic pain and infertility in reproductive‐aged women and is associated with dysmenorrhea, dyspareunia, dyschezia, and dysuria.^1^ About 10% of reproductive‐age women and 5%–50% of women with infertility experience endometriosis.^2^ The aetiology of endometriosis, which is considered to be multifactorial, remains largely elusive; nevertheless, the most well‐accepted theory for the pathogenesis of endometriosis is Sampson's theory.^3^ In which, viable endometrial fragments refluxed into the peritoneal cavity during menstruation, can implant, develop, and invade other tissues of the pelvis.^3^ Following retrograde menstruation, immune dysfunction has been theorized to facilitate successful lesion development after the displacement of endometrial tissue into ectopic sites.^4^ Numerous cytokines and growth factors, such as monocyte chemotactic protein‐1 (MCP‐1),^5^, ^6^, ^7^ hepatocyte growth factor (HGF),^5^, ^8^ and insulin‐like growth factor‐1 (IGF‐1)^9^, ^10^ have been shown to be elevated in peritoneal fluid (PF) of women with endometriosis. We also recently showed increased MCP‐1, HGF, and IGF‐1 serum and PF levels in endometriotic patients compared with controls.^11^ MCP‐1 affects endometriosis development by promoting proliferation and activating and recruiting mononuclear cells to secrete growth factors and cytokines.^12^, ^13^ HGF as a pleiotropic growth factor, can be involved in endometriosis development via its mitogenic, angiogenic, motogenic (migration), and morphogenic activities,^14^ and IGF‐1, as another growth factor, exerts its effect on endometriosis development by stimulating the growth and preventing apoptosis of endometrial cells.^15^, ^16^ Despite decades of research, treatment of endometriosis is currently limited to hormonal therapy or surgery which are non‐curative and frequently lead to endometriosis recurrence after cessation of treatment.^17^ Thus, recently, there has been an increasing emphasis on finding naturally occurring compounds for managing endometriosis. One of these compounds which has considerable anti‐inflammatory, anti‐proliferative, and even immunomodulatory effects, is vitamin D.^18^ Studies indicate that vitamin D influences women's reproductive health. Ectopic endometrium in endometriotic women, as well as endometriotic stromal cells, were shown to express 1alpha‐hydroxylase (1α‐OHase), which activates 25‐hydroxyvitamin D3 (25(OH)D3).^19^ A recent systematic review and meta‐analysis showed lower 25(OH)D3 serum levels in endometriosis women compared with controls. Also, a negative correlation between vitamin D levels and the severity of endometriosis was observed in that study.^20^ We also showed lower serum and PF levels of 25(OH)D3 in the patients with endometriosis compared with controls.^21^ Regarding 1,25‐dihydroxyvitamin D3 (1,25(OH)2D3) serum concentration results were controversial.^22^ The genomic pathway responsible for vitamin D activity is regulated by vitamin D receptor (VDR), which is expressed in many tissues and numerous tumours.^23^ Based on Agic et al.'s findings, VDR expression in the endometrium of endometriotic women lay between the level seen in ovarian cancer and control groups.^24^ Vitamin D can regulate the entire process of tumorigenesis through mechanisms such as proliferation, differentiation, apoptosis, migration, invasion, inflammation, and oxidative stress,^25^ and based on findings of a recent systematic review, adequate vitamin D levels were associated with a lower risk of ovarian cancer and reduced cancer mortality in the general population.^26^ Besides, recent studies pointed to the protective effects of vitamin D on endometriotic lesions^27^, ^28^ and also endometriotic stromal cells.^29^, ^30^

Considering the importance of MCP‐1, HGF, and IGF‐1 in the development of endometriosis and the inhibitory effect of vitamin D on the expression of these factors in other cell types in different diseases, we proposed a hypothesis that vitamin D may have an inhibitory effect on the development of endometriosis by reducing MCP‐1, HGF, and IGF‐1 expression in peripheral blood mononuclear cells (PBMCs), peritoneal fluid mononuclear cells (PFMCs), and endometrial stromal cells (ESCs) of women with endometriosis compared with non‐endometriotic patients. In this study, for the first time, we sought to investigate the effect of the active form of vitamin D on the expression of these factors at gene and protein level in PBMCs, PFMCs, and ESCs of women with endometriosis compared with non‐endometriotic patients. Besides, to find the anti‐proliferative effects of 1,25(OH)2D3, we evaluated the effect of 1,25(OH)2D3 on the proliferation of PBMCs and PFMCs of patients with and without endometriosis.

## 2 MATERIALS AND METHODS

### 2.1 Participants

This study included 45 patients admitted to the gynaecology ward of Rassoul‐Akram hospital. Of these, 30 women with endometriosis (stage III‐IV) were designated as the experimental group and 15 women with benign gynaecological diseases and no evidence of endometriosis were selected as the control group. All women enrolled were at reproductive age (24–45 years old) and were at the proliferative phase of the menstrual cycle. None of the included patients had pelvic inflammatory disease, adenomyosis, malignancy or autoimmune diseases and no patient took immunosuppressive and hormonal treatment or vitamin D3 supplement within 6 months before surgery. The diagnosis of endometriosis was initially evaluated by a clinician during laparoscopy and then confirmed by histopathological examination. The extent and severity of endometriosis were graded according to the revised American Society for Reproductive Medicine (rASRM).^31^ The study protocol was approved by the Ethics Committee of Iran University of Medical Sciences (Code: IR.IUMS.REC 1394.26098) and written informed consent was obtained from all patients. All methods were performed in accordance with the relevant guidelines and regulations.

### 2.2 Sample collection

Peripheral blood samples were collected in ethylenediaminetetraacetic acid (EDTA) Falcon tubes under sterile conditions from both groups before the administration of general anaesthesia and PF samples were collected before any operative manipulation to minimize blood contamination.

Ectopic lesions and eutopic endometrial tissues were collected from the same participant (endometriotic patient) using laparoscopy and biopsy curette, respectively. Eutopic endometrium also was collected from non‐endometriotic women. In the case of virgin patients, only ectopic lesions were collected. Tissue samples were immediately placed in Dulbecco's modified Eagle's medium (DMEM)‐F12 (Gibco) supplemented with 1% penicillin–streptomycin (pen‐strep) antibiotics (Gibco) and transferred under sterile conditions to the laboratory. For confirmation of endometriosis, a portion of the endometrial tissue was sent to the pathological laboratory.

Some samples were missed owning to culture contamination, obtaining the undesired cells, inappropriate pathology reports, and low peritoneal cell count which were caused by low volume of PF and gross bloody PF. At the end, 10 peripheral blood, 8 PF, 10 eutopic and 8 ectopic endometrial tissues from 30 endometriotic patients and 10 peripheral blood, 8 PF, and 10 eutopic endometrial tissues from 15 non‐endometriotic patients were used in this study.

### 2.3 Mononuclear cell culture

Peripheral blood mononuclear cells and PFMCs were separated, respectively, from blood and PF samples using density gradient centrifugation with Ficoll–Hypaque (Sigma‐Aldrich). About 1 × 10^6^ cells/ml of PBMCs and PFMCs were cultured in Roswell Park Memorial Institute medium (RPMI‐1640; Gibco) supplemented with 5% charcoal‐stripped foetal bovine serum (CS‐FBS; Sigma‐Aldrich) and 1% pen‐strep (Gibco).

### 2.4 ESC isolation procedure and cell culture conditions

Isolation, culture, and purification of ESCs were explained earlier.^32^ Briefly, endometrial tissues obtained from patients and controls were cut into 1 mm^3^ pieces and digested in DMEM‐F12 (Gibco) containing penicillin (100 U/ml) and streptomycin (100 μg/ml; Gibco), collagenase type I (2 mg/ml; Sigma‐Aldrich) and deoxynuclease I (300 μg/ml; Takara) in a humidified 5% CO_2_ at 37°C for 2 h with intermittent vortexing every 15 min. Following the removal of the undigested tissue using 100 μm mesh (BD Biosciences), cells were cultured in DMEM‐F12 (Gibco) medium supplemented with 10% CS‐FBS (Sigma‐Aldrich) and 1% pen‐strep antibiotic (Gibco) in a humidified 5% CO_2_ at 37°C for 24 h. After the removal of non‐adherent cells by washing with warm medium, adherent stromal cells were allowed to multiply. To evaluate the purity of ESCs, immunofluorescent staining and flow cytometry analysis were used. These cells were characterized as a panel of vimentin^+^, nestin^+^, cytokeratin^−^, CD10^+^, CD44^+^, CD73^+^, CD105^+^, CD34^−^, and CD45^−^ cells.^33^

### 2.5 Treatment of PBMCs and PFMCs, and ESCs with 1,25(OH)2D3

About 1 × 10^6^ PBMCs and PFMCs from each participant were seeded in each well of 24‐well plates and were treated with 1,25(OH)2D3 or ethanol at the 0.1 μM concentration.^34^ Also, about 1.6 × 10^5^ ESCs were seeded in each well of 12‐well plate and were treated with pre‐optimized 1,25(OH)2D3 (0.1 μM) or ethanol as a vehicle.^29^ This concentration of 1,25(OH)2D3 is equivalent to the physiologic level of this hormone.^35^ Seventy‐two h later, PBMCs were stimulated with ionomycin (1 μg/ml) and phorbol 12‐myristate‐13‐acetate (PMA; 50 ng/ml; Sigma‐Aldrich) and ESCs were stimulated with the lipopolysaccharide (LPS; 100 ng/ml; Sigma‐Aldrich).^36^, ^37^ Then, cells were incubated for three time points 6, 24, and 48 h.

### 2.6 Total RNA extraction, complementary DNA (cDNA) synthesis, and quantitative real‐time PCR (qRT‐PCR)

Total RNA was extracted from PBMCs, PFMCs, and ESCs using Trizol solution (Qiagen) based on the manufacturer's protocol. Quantity and purity of the extracted RNA were measured using a NanoDrop 2000 spectrophotometer (Thermo Fisher Scientific), and RNA integrity was assessed by electrophoresis on 2% agarose gel. For cDNA synthesis, 1 μg of RNA was reverse transcribed into cDNA using a Revert Aid First Strand cDNA Synthesis Kit (Thermo Fisher Scientific) according to the protocol. The qRT‐PCR was performed in duplicate using Rotor‐Gene 3000 (Corbett Research) with the SYBER premix Extaq (Biofact). The mRNA expression of *MCP‐1*, *HGF*, and *IGF‐1* were normalized using glyceraldehyde 3‐phosphate dehydrogenase (*GAPDH*) mRNA as a housekeeping gene. The sequences of the primers and size of amplicons are shown in Table 1. Briefly, 10 μl SYBER premix Extaq (Biofact), 1 μl primer pairs, 1 μl cDNA template, and 8 μl DNase‐free water were amplified in Rotor‐Gene 3000 with cycling conditions as 95°C step for 15 min (initial denaturation and activation of enzyme), followed by 40 cycles of 95°C for 20 s, annealing and elongation at 60°C for 40 s and the melting step at 60 to 99°C. All reactions were run in duplicate.

**TABLE 1:** The MCP‐1, HGF, IGF‐1, and GAPDH primers sequences.

Gene	Forward Primer	Reverse Primer	Amplicon size (bp)
MCP‐1	*5′‐GAAAGTCTCTGCCGCCCTT‐3′*	*5′‐TTGATTGCATCTGGCTGAGCG‐3′*	84
HGF	*5′‐GCAATTAAAACATGCGCTGACA‐3′*	*5′‐TCCCAACGCTGACATGGAAT‐3′*	140
IGF‐1	*5′‐CTCTTCAGTTCGTGTGTGGAGAC‐3′*	*5′‐CAGCCTCCTTAGATCACAGCTC‐3′*	134
GAPDH	*5′‐GCACCGTCAAGGCTGAGAAC‐3′*	*5′‐TGGTGAAGACGCCAGTGGA‐3′*	138

Abbreviations: bp, Base pair; GAPDH, Glyceraldehyde 3‐phosphate dehydrogenase; HGF, Hepatocyte growth factor; IGF‐1, Insulin‐like growth factor‐1; MCP‐1, Monocyte chemoattractant protein‐1.

### 2.7 Enzyme‐linked immunoassay (ELISA) procedure

The concentrations of MCP‐1, HGF, and IGF‐1 proteins were measured in PBMCs, PFMCs, and ESCs supernatant by a standard ELISA kit (Duoset; R&D Systems) based on the manufacturer's protocol. The absorbance was measured at 570 nm by a microplate reader (Bio‐Rad). The detection limit for MCP‐1, HGF, and IGF‐1 were 15.6–1000, 125.0–8000, and 31.2–2000 pg/ml, respectively.

### 2.8 Carboxyfluorescein diacetate, succinimidyl ester (CFSE)

Cell proliferation was evaluated using CFSE assay. Briefly, PBMCs and PFMCs were stained with 5 μM CFSE in 1 ml phosphate‐buffered saline (PBS; Biolegend) for 20 min in a CO_2_ incubator at 37°C and 5% CO_2_ (keep protected from the light). For quenching the staining and removal of the remaining free dye, cells were incubated with 5 ml complete growth medium for 10 min at 37°C and 5% CO_2_. The cells were washed three times and resuspended in the complete growth medium, then treated with 1,25(OH)2D3. After 72 h, cells were stimulated with ionomycin (1 μg/ml) and PMA (50 ng/ml; Sigma‐Aldrich). Five days later, PBMCs and PFMCs were harvested and cell proliferation was analysed on BD FACSCalibur. The flow cytometry data were analysed using the software FlowJo (Tree Star version 10.1r5 Inc.).

### 2.9 Statistical analysis

Statistical analyses were performed using the GraphPad Prism software 8 (GraphPad Software, Inc.). Kolmogorov–Smirnov test was applied to assess the normal distribution of data. All data were analysed using the non‐parametric tests, including the Wilcoxon signed‐rank test, Mann–Whitney, and Kruskal‐Wallis tests. The mRNA expression analysis was performed using the 2^−ΔΔCt^ method. A *p*‐value of <0.05 was considered statistically significant.

## 3 RESULTS

### 3.1 The effect of 1,25(OH)2D3 treatment on MCP‐1 gene and protein expression by PBMCs, PFMCs, and ESCs

**FIGURE 1:**
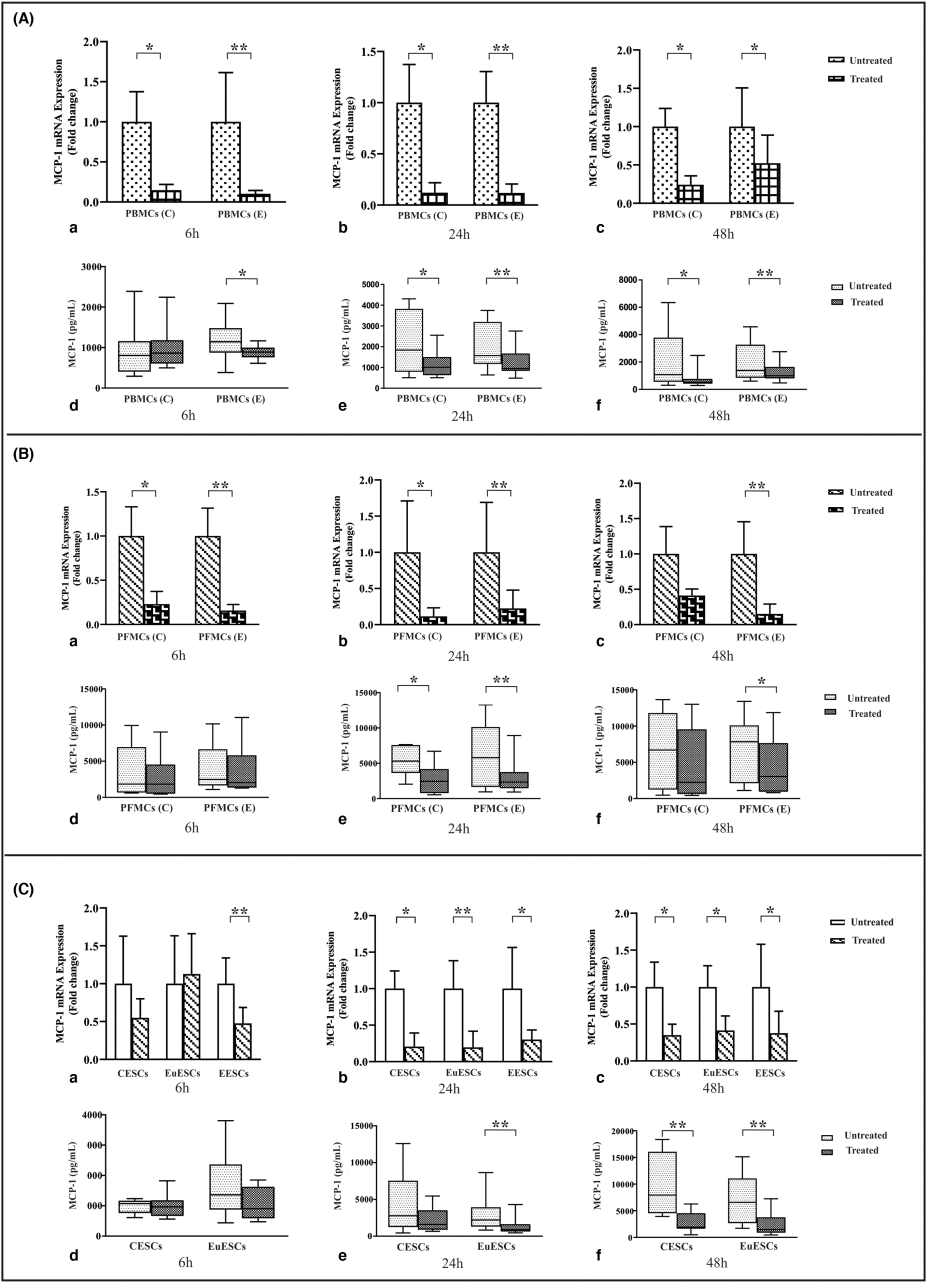
The gene and protein expression of MCP‐1 by PBMCs, PFMCs, and ESCs after treatment with 1,25(OH)2D3. PBMCs of endometriosis patients and control participants (*n* = 10), PFMCs of endometriosis patients and control participants (*n* = 8), EESCs (*n* = 8), EuESCs (*n* = 10), and CESCs (*n* = 10) were treated with 0.1 μM 1,25(OH)2D3 at 6, 24, and 48 h. Results were analysed using a non‐parametric test. (A) Treatment of PBMCs, (B) Treatment of PFMCs, and (C) Treatment of ESCs. (a) The gene expression of *MCP‐1* at 6 h (D_3_ +/−), (b) The gene expression of *MCP‐1* at 24 h (D_3_ +/−), (c) The gene expression of *MCP‐1* at 48 h (D_3_ +/−), (d) protein production of MCP‐1 at 6 h (D_3_ +/−), (e) protein production of MCP‐1 at 24 h (D_3_ +/−), and (f) protein production of MCP‐1 at 48 h (D_3_ +/−). Data were represented as mean ± SEM and min to max. *p < 0.05 and **p < 0.01. CESCs, control endometrial stromal cells; EESCs, ectopic endometrial stromal cells; ESCs, endometrial stromal cells; EuESCs, eutopic endometrial stromal cells; MCP‐1, monocyte chemoattractant protein‐1; PBMCs, peripheral blood mononuclear; PFMCs, peritoneal fluid mononuclear cells

Figure 1 shows the data obtained from the MCP‐1 gene and protein expression in PBMCs, PFMCs, and ESCs treated with 1,25(OH)2D3 after 6, 24, and 48 h. 1,25(OH)2D3 treatment significantly reduced *MCP‐1* gene expression in PBMCs of patients with and without endometriosis compared with untreated controls at 6 and 24 h (*p* < 0.01 and <0.05, respectively) and 48 h (*p* < 0.05; Figure 1Aa,b,c). Regarding protein expression, 1,25(OH)2D3 treatment decreased MCP‐1 expression in PBMCs of endometriosis patients in all time intervals (*p* < 0.05–<0.01; Figure 1Ad,e,f). Also, 1,25(OH)2D3 treatment decreased MCP‐1 protein expression in PBMCs of non‐endometriotic participants at 24 and 48 h (*p* < 0.05; Figure 1Ae,f).

Gene expression analysis revealed that 1,25(OH)2D3 significantly decreased the expression levels of *MCP‐1* in PFMCs of patients with and without endometriosis compared with untreated control at 6 and 24 h of treatment (*p* < 0.01 and <0.05, respectively; Figure 1Ba,b). But, 1,25(OH)2D3 treatment significantly reduced gene expression of *MCP‐1* in PFMCs of endometriotic patients at 48 h (*p* < 0.01; Figure 1Bc). Regarding protein expression, 1,25(OH)2D3 treatment significantly reduced MCP‐1 expression at 24 and 48 h in PFMCs of endometriotic patients (*p* < 0.01 and <0.05, respectively; Figure 1Be,f). 1,25(OH)2D3 treatment significantly reduced MCP‐1 protein expression in PFMCs at 24 h in control participants (*p* < 0.05; Figure 1Be).

Vitamin D treatment reduced *MCP‐1* gene expression in ectopic endometrial stromal cells (EESCs) at 6 h (*p* < 0.01; Figure 1Ca). Also, 1,25(OH)2D3 treatment significantly reduced *MCP‐1* gene expression in control endometrial stromal cells (CESCs), eutopic endometrial stromal cells (EuESCs), and EESCs at 24 and 48 h (*p* < 0.05–<0.01; Figure 1Cb,c). Regarding protein expression, 1,25(OH)2D3 treatment significantly reduced MCP‐1 expression at 24 and 48 h in EuESCs of endometriotic patients (*p* < 0.01; Figure 1Ce,f) and at 48 h in CESCs (*p* < 0.01; Figure 1Cf).

### 3.2 The effect of 1,25(OH)2D3 treatment on HGF gene and protein expression by PBMCs, PFMCs, and ESCs

**FIGURE 2:**
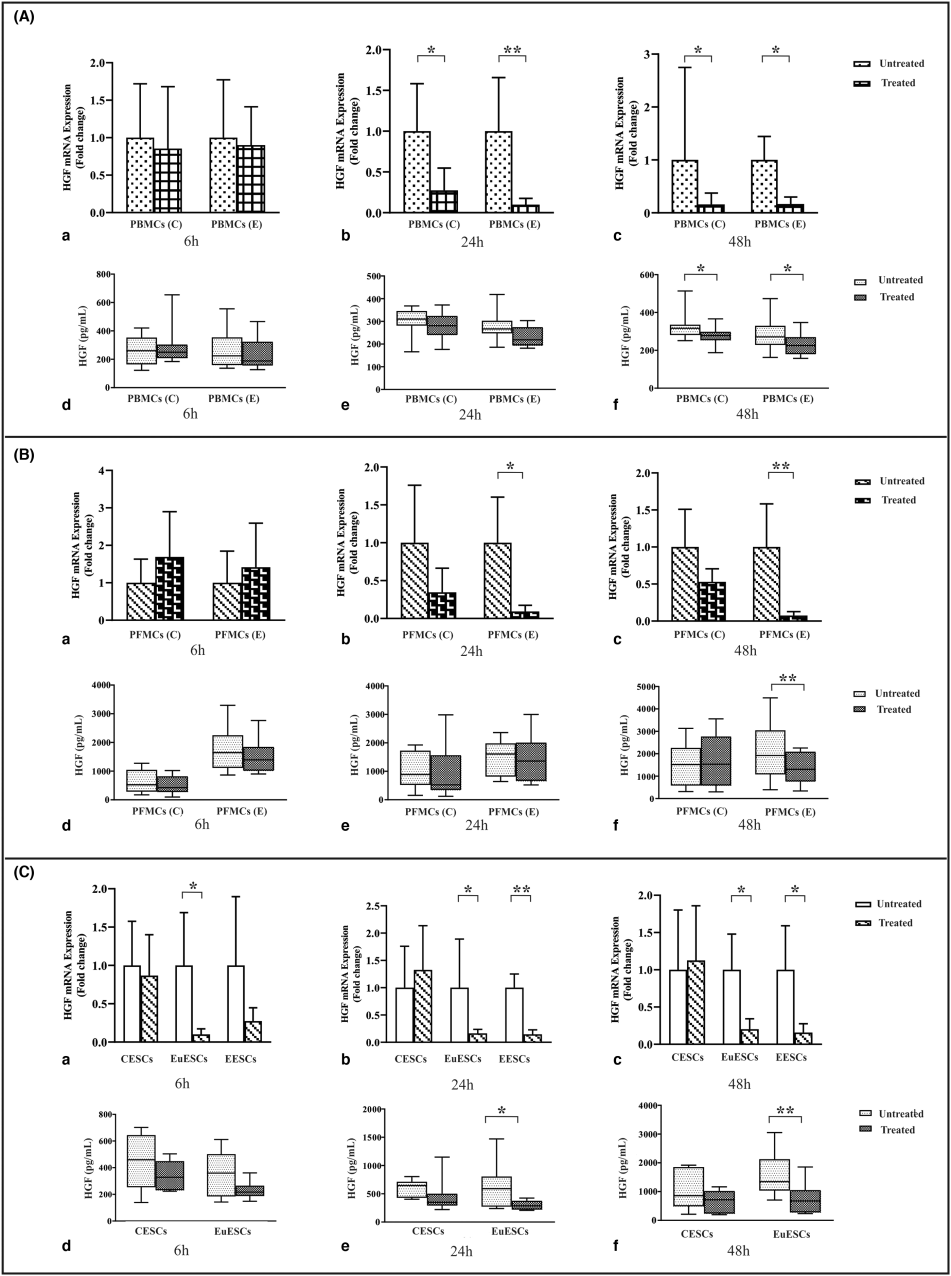
The gene and protein expression of HGF by PBMCs, PFMCs, and ESCs after treatment with 1,25(OH)2D3. PBMCs of endometriosis patients and control participants (*n* = 10), PFMCs of endometriosis patients and control participants (*n* = 8), EESCs (*n* = 8), EuESCs (*n* = 10), and CESCs (*n* = 10) were treated with 0.1 μM 1,25(OH)2D3 at 6, 24, and 48 h. Results were analysed using a non‐parametric test. (A) Treatment of PBMCs, (B) Treatment of PFMCs, and (C) Treatment of ESCs. (a) The gene expression of *HGF* at 6 h (D_3_ +/−), (b) The gene expression of *HGF* at 24 h (D_3_ +/−), (c) The gene expression of *HGF* at 48 h (D_3_ +/−), (d) protein production of HGF at 6 h (D_3_ +/−), (e) The protein production of HGF at 24 h (D_3_ +/−), and (f) The protein production of HGF at 48 h (D_3_ +/−). Data were represented as mean ± SEM and min to max. *p < 0.05 and **p < 0.01. CESCs, control endometrial stromal cells; EESCs, ectopic endometrial stromal cells; ESCs, endometrial stromal cells; EuESCs, eutopic endometrial stromal cells; HGF, Hepatocyte growth factor; PBMCs, peripheral blood mononuclear; PFMCs, peritoneal fluid mononuclear cells

1,25(OH)2D3 treatment had no significant effect on *HGF* gene expression in PBMCs of patients with and without endometriosis compared with untreated controls at 6 h (Figure 2Aa).

The gene expression of *HGF* in PBMCs of patients with and without endometriosis after 1,25(OH)2D3 treatment compared with untreated controls showed a significant reduction after 24 h (*p* < 0.01 and <0.05, respectively; Figure 2Ab). 1,25(OH)2D3 treatment significantly reduced HGF gene and protein expression in PBMCs of endometriotic and non‐endometriotic patients at 48 h (*p* < 0.05; Figure 2Ac,f). This treatment had no significant effect on HGF protein expression in PBMCs of patients with and without endometriosis compared with untreated controls at 6 and 24 h (Figure 2Ad,e). Regarding PFMCs, 1,25(OH)2D3 treatment increased *HGF* gene expression in PFMCs of endometriotic and non‐endometriotic patients compared with untreated controls at 6 h but that was non‐significant (Figure 2Ba).

1,25(OH)2D3 treatment reduced *HGF* gene expression in the PFMCs of endometriosis patients at 24 and 48 h (*p* < 0.05 and <0.01, respectively; Figure 2Bb,c). 1,25(OH)2D3 treatment had no significant effect on HGF protein expression in PFMCs of patients with and without endometriosis compared with untreated controls at 6 and 24 h (Figure 2Bd,e) while this treatment reduced HGF protein expression in the PFMCs of endometriosis patients at 48 h (*p* < 0.01; Figure 2Bf). Vitamin D treatment reduced *HGF* gene expression in EuESCs in all time intervals (*p* < 0.05; Figure 2Ca,b,c). Also, 1,25(OH)2D3 treatment significantly reduced *HGF* gene expression in EESCs at 24 and 48 h (*p* < 0.01 and <0.05, respectively; Figure 2Cb,c). Regarding protein expression, 1,25(OH)2D3 treatment had no significant effect on HGF protein expression in EuESCs and CESCs at 6 h (Figure 2Cd) while this treatment significantly reduced HGF protein expression at 24 and 48 h in EuESCs of endometriotic patients (*p* < 0.05 and <0.01, respectively; Figure 2Ce,f).

### 3.3 The effect of 1,25(OH)2D3 treatment on IGF‐1 gene and protein expression by PBMCs, PFMCs, and ESCs

**FIGURE 3:**
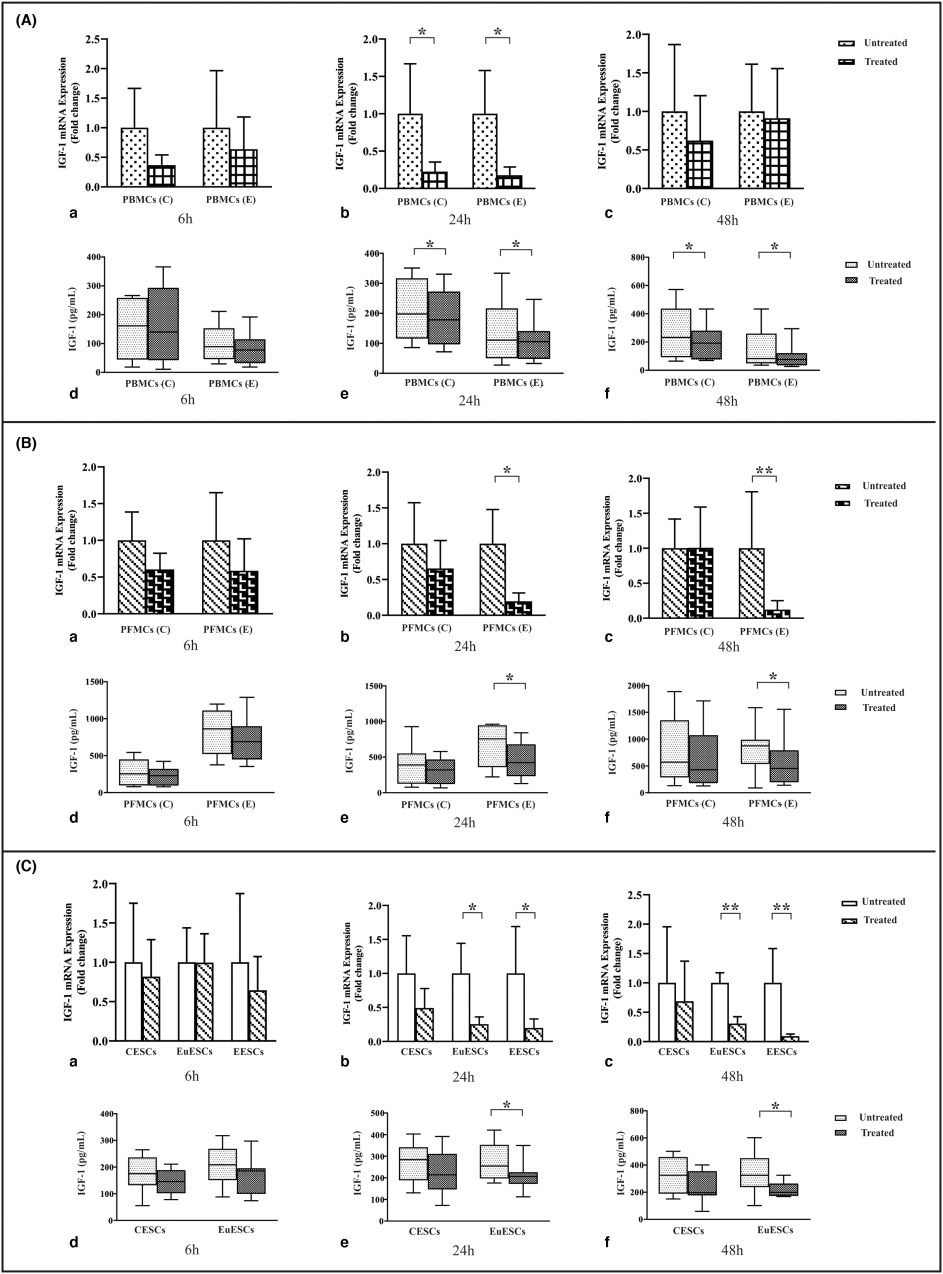
The gene and protein expression of IGF‐1 by PBMCs, PFMCs, and ESCs after treatment with 1,25(OH)2D3. PBMCs of endometriosis patients and control participants (*n* = 10), PFMCs of endometriosis patients and control participants (*n* = 8), EESCs (*n* = 8), EuESCs (*n* = 10), and CESCs (*n* = 10) were treated with 0.1 μM 1,25(OH)2D3 at 6, 24, and 48 h. Results were analysed using a non‐parametric test. (A) Treatment of PBMCs, (B) Treatment of PFMCs, and (C) Treatment of ESCs. (a) The gene expression of *IGF‐1* at 6 h (D_3_ +/−), (b) The gene expression of *IGF‐1* at 24 h (D_3_ +/−), (c) The gene expression of *IGF‐1* at 48 h (D_3_ +/−), (d) protein production of IGF‐1 at 6 h (D_3_ +/−), (e) protein production of IGF‐1 at 24 h (D_3_ +/−), and (f) protein production of IGF‐1 at 48 h (D_3_ +/−). Data were represented as mean ± SEM and min to max. *p < 0.05 and **p < 0.01. CESCs, control endometrial stromal cells; EESCs, ectopic endometrial stromal cells; ESCs, endometrial stromal cells; EuESCs, eutopic endometrial stromal cells; IGF‐1, Insulin growth factor‐1; PBMCs, peripheral blood mononuclear; PFMCs, peritoneal fluid mononuclear cells

1,25(OH)2D3 treatment had no significant effect on *IGF‐1* gene expression in PBMCs of patients with and without endometriosis compared with untreated controls at 6 h (Figure 3Aa).

1,25(OH)2D3 treatment reduced the gene expression of *IGF‐1* in PBMCs of endometriosis patients and non‐endometriotic individuals at 24 h (*p* < 0.05; Figure 3Ab). Such treatment did not significantly change *IGF‐1* gene expression in PBMCs of patients with and without endometriosis compared with untreated controls at 48 h (Figure 3Ac). Also this treatment had no significant effect on IGF‐1 protein expression in PBMCs of these two groups compared with untreated controls at 6 h (Figure 3Ad) while the protein expression of IGF‐1 in PBMCs of endometriosis patients and non‐endometriotic individuals decreased significantly at 24 and 48 h following 1,25(OH)2D3 treatment (*p* < 0.05; Figure 3Ae,f). However, the reducing effect of vitamin D treatment on IGF‐1 protein expression was more remarkable in PBMCs of endometriosis patients at 24 h (*p* < 0.05; data not shown).

1,25(OH)2D3 treatment did not significantly change *IGF‐1* gene expression in PFMCs of endometriotic and non‐endometriotic patients compared with untreated controls at 6 h (Figure 3Ba). Gene expression of *IGF‐1* in PFMCs of endometriosis patients showed a significant decrease after 24 and 48 h following 1,25(OH)2D3 treatment (*p* < 0.05 and <0.01, respectively; Figure 3Bb,c). Such treatment had no significant effect on IGF‐1 protein expression in PFMCs of these two groups compared with untreated controls at 6 h (Figure 3Bd). Protein expression of IGF‐1 also decreased significantly in PFMCs of endometriosis patients after 24 and 48 h following 1,25(OH)2D3 treatment (*p* < 0.05; Figure 3Be,f). 1,25(OH)2D3 treatment did not significantly change *IGF‐1* gene expression in CESCs, EuESCs, and EESCs at 6 h (Figure 3Ca). 1,25(OH)2D3 treatment decreased *IGF‐1* gene expression in EuESCs and EESCs at 24 (*p* < 0.05; Figure 3Cb) and 48 h (*p* < 0.01; Figure 3Cc). Such treatment had no significant effect on IGF‐1 protein expression in CESCs and EuESCs at 6 h (Figure 3Cd) while this treatment decreased IGF‐1 protein expression in EuESCs at 24 and 48 h (*p* < 0.05; Figure 3Ce,f).

### 3.4 The effect of 1,25(OH)2D3 treatment on cell proliferation

**FIGURE 4:**
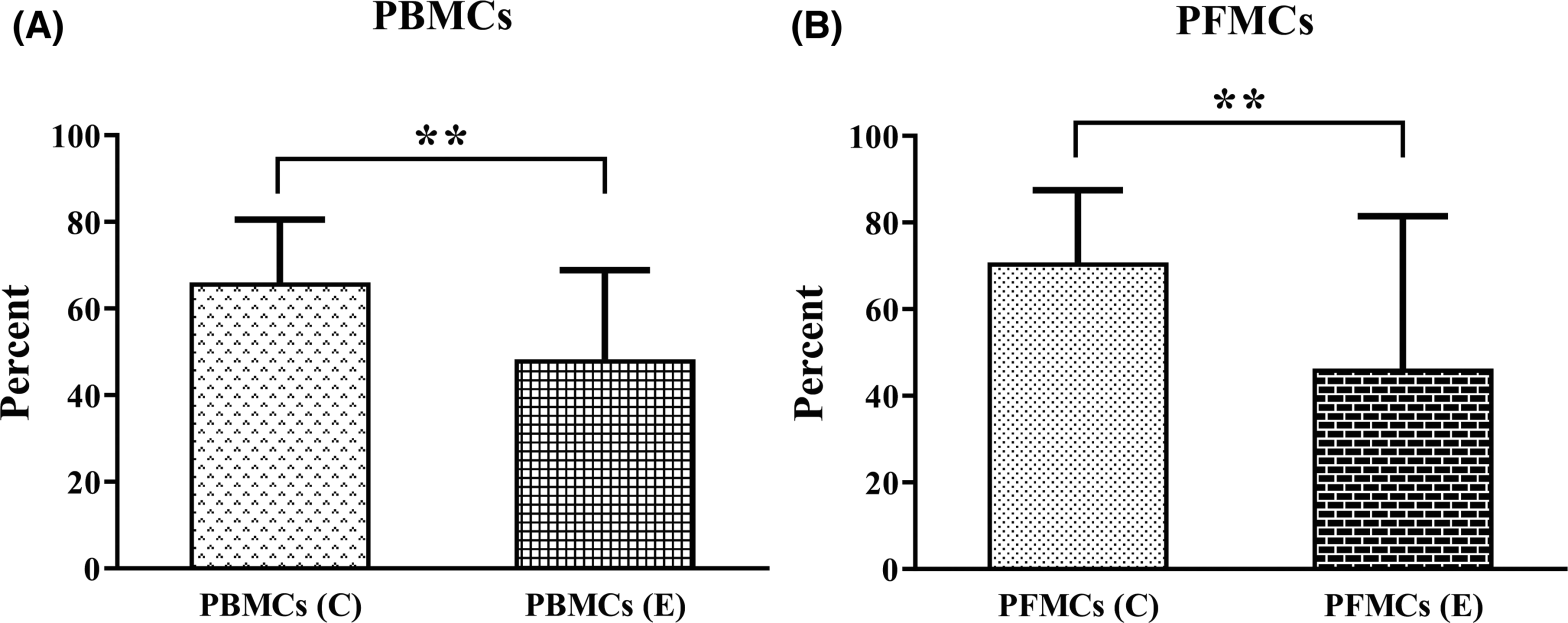
(A) The proliferation rate of PBMCs and (B) PFMCs of endometriosis and control participants after 1,25(OH)2D3 treatment. Results reported as mean ± SD. PBMCs of endometriosis patients and control participants (*n* = 10), PFMCs of endometriosis patients and control participants (*n* = 8). **p < 0.01. PBMCs, peripheral blood mononuclear cells; PFMCs, peritoneal fluid mononuclear cells

Data obtained from the CFSE assay showed that 1,25(OH)2D3 can reduce the proliferation of PBMCs and PFMCs of endometriosis patients (*p* < 0.01, for both cell types; Figure 4).

## 4 DISCUSSION

In this study, 1,25(OH)2D3 treatment significantly reduced the protein expression of MCP‐1, HGF, and IGF‐1 in PBMCs and PFMC of endometriosis patients at 48 h. Also, 1,25(OH)2D3 treatment significantly reduced *MCP‐1*, *HGF*, and *IGF‐1* gene expression in EESCs at 24 and 48 h, and such treatment reduced gene and protein expression of mentioned factors at 24 and 48 h in EuESCs. Also, 1,25(OH)2D3 treatment, in our study, significantly inhibited the proliferation in PBMCs and PFMCs of endometriotic patients compared with controls. According to our knowledge, this study is the first study to investigate the effect of 1,25(OH)2D3, the active form of vitamin D, treatment on MCP‐1, HGF, and IGF‐1 gene and protein expression in PBMCs, PFMCs, and ESCs of patients with and without endometriosis.

A unifying theory regarding the origin of endometriosis has not been precisely understood. However, the retrograde menstruation theory of Sampson is the most widely accepted one.^3^ Reflux menstruation of endometrial tissue is routinely observed in almost all reproductive‐aged women, but only ∼10%–20% of them develop endometriosis. Therefore, it is plausible that other mechanisms like dysregulated immunity or inflammatory markers might act in unison to cause endometriosis.^4^ On the basis of previous studies, the critical role of chemokines and growth factors are well defined in relation to the pathogenesis of endometriosis in which chemokines, such as MCP‐1 and growth factors, such as HGF and IGF‐1 have been shown to be elevated in the serum^11^, ^38^, ^39^ and PF of women with endometriosis.^5^, ^10^, ^11^, ^39^

MCP‐1 (CCL2) and its receptor CC motif chemokine receptor‐2 (CCR2) play a key role in endometriosis initiation and development.^40^ MCP‐1 is produced by different cells, including macrophages, fibroblasts, and endometriotic stromal cells.^41^, ^42^ MCP‐1 promotes monocyte migration from peripheral blood to the peritoneal cavity where they transform into macrophages and cause local inflammation in the peritoneal cavity.^12^ Moreover, in a study by Li et al. recombinant human CCL2 in the EuESCs promoted survivin and matrix metalloproteinase 2 (MMP2) expression and stimulated ESCs proliferation, viability, and invasion through activation of Akt and mitogen‐activated protein kinase (MAPK)/extracellular signal‐regulated kinase (Erk)1/2 signalling pathway while both anti‐CCL2 neutralizing antibody and CCR2 antagonist abolished these effects.^13^ On the basis of our recent findings, gene and/or protein expression of MCP‐1 by PBMCs and PFMCs was higher in endometriotic women compared with controls.^11^ Furthermore, based on a recent study, EESCs expressed more MCP‐1 gene and/or protein compared with EuESCs and CESCs.^11^, ^32^ According to these findings, MCP‐1 may be involved in the pathogenesis of endometriosis. The present study is the first to investigate the effect of 1,25(OH)2D3 treatment on MCP‐1 expression in PBMCs, PFMCs, and ESCs of patients with endometriosis compared with controls. Treatment with 1,25(OH)2D3 in our study significantly reduced gene and protein expression of MCP‐1 in PBMCs and PFMCs of endometriotic patients at 24 and 48 h. Also, this treatment reduced *MCP‐1* gene and/or protein expression in EESCs, EuESCs, and CESCs. Consistent with our findings, 1,25(OH)2D3 treatment in other studies with different cell types significantly reduced MCP‐1 secretion.^43^, ^44^, ^45^

HGF, also known as the scatter factor, is an important growth factor related to endometriosis.^14^ The HGF receptor is the c‐met proto‐oncogene product (c‐Met).^14^ Peritoneal macrophages appear to be the major source of most endometriosis‐related cytokines. But regarding HGF, the peritoneum and endometriotic stromal cells seem to be primary production sources of this cytokine in endometriosis.^46^ Inflammatory cytokines such as IL‐6, LPS, and prostaglandins known as HGF inducers stimulate HGF production in the pelvic cavity of patients with endometriosis.^46^ We recently showed increased HGF gene and protein expression in PFMCs of endometriotic patients compared with controls.^11^ Overexpression of HGF in ectopic lesions compared with eutopic endometrium of endometriotic patients has been shown in one study.^47^ Besides, Khan et al. showed increased HGF and c‐Met immunoexpressions in the eutopic endometrium of endometriotic patients than in the controls.^48^ Regarding ESCs, recent studies revealed higher *HGF* gene expression in EESCs compared with EuESCs^11^, ^33^, ^49^ and Sugawara et al. showed upregulation in the secretion of HGF in EuESCs compared with CESCs.^50^ HGF‐Met system was shown to promote stromal cell proliferation and invasion of shed eutopic and ectopic endometrium through autocrine and paracrine pathways.^46^ Besides, based on a recent study, HGF can be used as the biomarkers for diagnosing endometriosis and predicting its prognosis.^51^ For the first time, 1,25(OH)2D3 treatment in our study significantly reduced gene and protein expression of HGF in PBMCs and PFMCs of endometriotic patients at 48 h. Also, this treatment reduced *HGF* gene expression in EESCs and HGF gene and protein expression in EuESCs at 24 and 48 h. Consistent with our results, 1,25(OH)2D3 treatment decreased HGF production in human promyelocytic leukaemia cell line^52^ and MG‐63 osteosarcoma cells.^53^

IGF‐1, which is synthesized by different cells, such as endometrial cells, prevents apoptosis and acts as a mitogen on ESCs cultured in vitro.^15^, ^16^ Studies on the association between IGF‐1 and endometriosis were inconsistent, but according to a recent prospective study, IGF‐1 and IGFBP‐3 were associated with a higher risk of endometriosis among younger women.^54^ Furthermore, it has been previously reported that endometriotic cysts significantly expressed lower levels of IGF‐I, both at the mRNA and protein levels compared with eutopic endometrium.^55^, ^56^ Contrary to these findings, in a study by Zhou et al. IGF‐I and IGF‐1R were expressed both in paired eutopic endometrium and ovarian endometrioma tissues.^57^ In addition, in that study, mRNA levels of *IGF‐I*, but not *IGF‐1R*, in EESCs were significantly higher than those in EuESCs.^57^ In line with these results, we recently showed higher gene and/or protein expression of IGF‐1 in EESCs compared with EuESCs and CESCs.^11^, ^33^ Besides, we showed higher gene and/or protein expression of IGF‐1 by PBMCs and PFMCs in endometriosis patients compared with controls.^11^ IGF‐I may contribute to endometriosis development via upregulation of oestrogen receptor beta (ERβ) and aromatase expression through IGF‐1R/ phosphatidylinositol 3‐kinase (PI3K)/AKT pathway^57^ so that inhibitors of this signalling pathway can suppress the development of endometriosis by downregulating the expression of pro‐inflammatory cytokines and proteolytic factors.^58^ For the first time, 1,25(OH)2D3 treatment in our study significantly reduced the protein expression of IGF‐1 in PBMCs and PFMCs of endometriotic patients at 24 and 48 h. Also, this treatment reduced *IGF‐1* gene expression in EESCs and IGF‐1 gene and protein expression in EuESCs at 24 and 48 h. Consistent with our findings, vitamin D treatment in breast cancer cell lines inhibited the mitogenic effects of IGF‐I,^59^ attenuated the antiapoptotic effects of IGF‐I^60^, ^61^ and downregulated the expression of IGF‐I receptors.^59^, ^61^

Endometriotic cells show higher endogenous oxidative stress levels because of excess reactive oxygen species (ROS) production and alterations in ROS detoxification pathways.^62^ Oxidative stress acts as a regulator of nuclear factor kappa B (NF‐κB) activation, which is involved in endometriosis onset and progression.^63^ In in vivo studies, constitutive activation of NF‐κB has been shown in ectopic endometriotic lesions and peritoneal macrophages of patients with endometriosis.^64^, ^65^ Increased levels of oxidative stress along with increased levels of pro‐inflammatory cytokines in endometriotic tissue would activate NF‐κB and activation of NF‐κB would further increase the production of chemokines and growth factors such as MCP‐1, HGF, and IGF‐1.^66^ So the suppression of NF‐κB activation may decrease proliferation and expression of these chemokines and growth factors in PBMCs, PFMCs, and ESCs of patients with endometriosis. On the basis of recent studies, 1,25(OH)2D3 as a powerful antioxidant has been shown to inhibit NF‐kB activation by increasing the stability of inhibitor of kappa B alpha (IkBα) protein in different cell types.^45^, ^67^, ^68^ Another mechanism by which vitamin D reduces inflammation and expression of these growth factors and chemokines in endometriosis may be through up‐regulation of mitogen‐activated protein kinase phosphatase‐1 (MKP‐1). MKP‐1 is known to preferentially inactivate p38 and c‐Jun N‐terminal kinase (JNK), leading to subsequent inhibition of pro‐inflammatory cytokines production.^69^ Besides, calcitriol has been shown to inhibit the growth of hepatocellular cell lines by down‐regulating c‐Met and extracellular signal‐regulated kinase (ERK) expression.^70^ Regarding IGF‐1, except anti‐mitogenic and anti‐apoptotic effects of vitamin D treatment on IGF‐1 levels in breast cancer cell line, vitamin D analogue EB1089 has been shown to inhibit the aromatase gene expression in breast cancer cells, via a VDR/Williams syndrome transcription factor (WSTF)‐mediated mechanism.^71^

Previously, we showed that the rate of the proliferation of EESCs and EuESCs significantly decreased after treatment with 1,25(OH)2D3 in the fibronectin‐coated plate.^29^ In the present study, 1,25(OH)2D3 treatment reduced the proliferation of PBMCs and PFMCs in endometriosis patients. In consistence with our data, previous studies showed that 1,25(OH)2D3 dose‐dependently reduced the proliferation of PBMCs stimulated by phytohaemagglutinin (PHA).^72^, ^73^ Studies have shown that in endometriosis the number and percentage of mononuclear cells (both in the blood and especially at the peritoneal cavity) increases.^74^ These cells increase the production of cytokines and chemokines (including MCP‐1, HGF, and IGF‐1). As a result, reducing the proliferation of these cells can reduce inflammation, thereby reducing the infiltration and proliferation of endometriotic cells.^74^

As limitation of the present study, we could not evaluate MCP‐1, HGF, and IGF‐1 protein expression in EESCs, because of the small number of EESCs that was due to the specific nature of EESCs and their difficult growth condition, so it should be examined in other studies.

Finally, considering the present and previous findings, 1,25(OH)2D3 seems to be a promising agent against endometriosis and thus it holds promise as a natural therapeutical agent, but further research is warranted based on encouraging in vitro and in vivo data.

## 5 CONCLUSIONS

Given the role of MCP‐1, HGF, and IGF‐1 in proliferation, invasion, and angiogenesis, it appears that these factors play vital roles in the proliferation of ESCs and their invasion into the extracellular matrix. Due to the reduction in the proliferation of PBMCs and PFMCs, as well as the reduced expression of these factors after treatment with 1,25(OH)2D3, it appears that this vitamin may play an important role in the reduction of inflammatory responses and the progression of the disease. According to the results of this study, 1,25(OH)2D3 can be used as an effective agent in the prevention and treatment of endometriosis along with other therapies.

## AUTHOR CONTRIBUTIONS

**Sahel Heidari:** Conceptualization (equal); formal analysis (equal); investigation (equal); methodology (equal); project administration (equal); writing – original draft (equal); writing – review and editing (equal). **Roya Kolahdouz‐Mohammadi:** Formal analysis (equal); writing – original draft (equal); writing – review and editing (equal). **Sepideh Khodaverdi:** Conceptualization (equal); writing – review and editing (equal). **Tahereh Mohammadi:** Project administration (equal); writing – review and editing (equal). **Ali‐Akbar Delbandi:** Conceptualization (equal); formal analysis (equal); investigation (equal); methodology (equal); project administration (equal); supervision (lead); writing – review and editing (equal).

## CONFLICT OF INTEREST

The authors confirm that there are no conflicts of interest.

## Data Availability

The data that support the findings of this study are available on request from the corresponding author. The data are not publicly available due to privacy or ethical restrictions.

## References

[1] Giudice LC . Endometriosis. N Engl J Med. 2010;362(25):2389‐2398. 20573927 10.1056/NEJMcp1000274 PMC3108065

[2] Shafrir AL , Farland LV , Shah DK , et al. Risk for and consequences of endometriosis: a critical epidemiologic review. Best Pract Res Clin Obstet Gynaecol. 2018;51:1‐15. 30017581 10.1016/j.bpobgyn.2018.06.001

[3] Sampson JA . Peritoneal endometriosis due to menstrual dissemination of endometrial tissue into the peritoneal cavity. Am J Obstet Gynecol. 1927;14:422‐469.

[4] Symons LK , Miller JE , Kay VR , et al. The immunopathophysiology of endometriosis. Trends Mol Med. 2018;24(9):748‐762. 30054239 10.1016/j.molmed.2018.07.004

[5] Jorgensen H , Hill AS , Beste MT , et al. Peritoneal fluid cytokines related to endometriosis in patients evaluated for infertility. Fertil Steril. 2017;107(5):1191‐1199 e2. 28433374 10.1016/j.fertnstert.2017.03.013

[6] Pizzo A , Salmeri FM , Ardita FV , Sofo V , Tripepi M , Marsico S . Behaviour of cytokine levels in serum and peritoneal fluid of women with endometriosis. Gynecol Obstet Invest. 2002;54(2):82‐87. 12566749 10.1159/000067717

[7] Bersinger NA , Dechaud H , McKinnon B , Mueller MD . Analysis of cytokines in the peritoneal fluid of endometriosis patients as a function of the menstrual cycle stage using the Bio‐Plex platform. Arch Physiol Biochem. 2012;118(4):210‐218. 22632541 10.3109/13813455.2012.687003

[8] Newaz Khan K , Masuzaki H , Fujishita A , et al. Peritoneal fluid and serum levels of hepatocyte growth factor may predict the activity of endometriosis. Acta Obstet Gynecol Scand. 2006;85(4):458‐466. 16612709 10.1080/00016340500432556

[9] Forster R , Sarginson A , Velichkova A , et al. Macrophage‐derived insulin‐like growth factor‐1 is a key neurotrophic and nerve‐sensitizing factor in pain associated with endometriosis. FASEB J. 2019;33(10):11210‐11222. 31291762 10.1096/fj.201900797R PMC6766660

[10] Kim JG , Suh CS , Kim SH , Choi YM , Moon SY , Lee JY . Insulin‐like growth factors (IGFs), IGF‐binding proteins (IGFBPs), and IGFBP‐3 protease activity in the peritoneal fluid of patients with and without endometriosis. Fertil Steril. 2000;73(5):996‐1000. 10785227 10.1016/s0015-0282(00)00493-3

[11] Heidari S , Kolahdouz‐Mohammadi R , Khodaverdi S , Tajik N , Delbandi AA . Expression levels of MCP‐1, HGF, and IGF‐1 in endometriotic patients compared with non‐endometriotic controls. BMC Womens Health. 2021;21(1):422. 34930225 10.1186/s12905-021-01560-6 PMC8686524

[12] Deshmane SL , Kremlev S , Amini S , Sawaya BE . Monocyte chemoattractant protein‐1 (MCP‐1): an overview. J Interferon Cytokine Res. 2009;29(6):313‐326. 19441883 10.1089/jir.2008.0027 PMC2755091

[13] Li M‐Q , Li H‐P , Meng Y‐H , et al. Chemokine CCL2 enhances survival and invasiveness of endometrial stromal cells in an autocrine manner by activating Akt and MAPK/Erk1/2 signal pathway. Fertil Steril. 2012;97(4):919‐929. 22265030 10.1016/j.fertnstert.2011.12.049

[14] Khan KN , Kitajima M , Hiraki K , et al. Immunopathogenesis of pelvic endometriosis: role of hepatocyte growth factor, macrophages and ovarian steroids. Am J Reprod Immunol. 2008;60(5):383‐404. 19238747 10.1111/j.1600-0897.2008.00643.x

[15] Giudice LC , Dsupin BA , Gargosky SE , Rosenfeld RG , Irwin JC . The insulin‐like growth factor system in human peritoneal fluid: its effects on endometrial stromal cells and its potential relevance to endometriosis. J Clin Endocrinol Metab. 1994;79(5):1284‐1293. 7525631 10.1210/jcem.79.5.7525631

[16] Koutsilieris M , Mastrogamvrakis G , Lembessis P , Sourla A , Miligos S , Michalas S . Increased insulin‐like growth factor 1 activity can rescue KLE endometrial‐like cells from apoptosis. Mol Med. 2001;7(1):20‐26. 11474124 PMC1949986

[17] Brown J , Farquhar C . An overview of treatments for endometriosis. JAMA. 2015;313(3):296‐297. 25603001 10.1001/jama.2014.17119

[18] Colonese F , Laganà AS , Colonese E , et al. The pleiotropic effects of vitamin D in gynaecological and obstetric diseases: an overview on a hot topic. Biomed Res Int. 2015;2015:986281. 26000308 10.1155/2015/986281 PMC4426767

[19] Vigano P , Lattuada D , Mangioni S , et al. Cycling and early pregnant endometrium as a site of regulated expression of the vitamin D system. J Mol Endocrinol. 2006;36(3):415‐424. 16720713 10.1677/jme.1.01946

[20] Qiu Y , Yuan S , Wang H . Vitamin D status in endometriosis: a systematic review and meta‐analysis. Arch Gynecol Obstet. 2020;302(1):141‐152. 32430755 10.1007/s00404-020-05576-5

[21] Delbandi A‐A , Torab M , Abdollahi E , et al. Vitamin D deficiency as a risk factor for endometriosis in Iranian women. J Reprod Immunol. 2021;143:103266. 33385732 10.1016/j.jri.2020.103266

[22] Kalaitzopoulos DR , Lempesis IG , Athanasaki F , et al. Association between vitamin D and endometriosis: a systematic review. Hormones (Athens). 2020;19(2):109‐121. 31863346 10.1007/s42000-019-00166-w

[23] Pike JW , Meyer MB , Lee S‐M , Onal M , Benkusky NA . The vitamin D receptor: contemporary genomic approaches reveal new basic and translational insights. J Clin Invest. 2017;127(4):1146‐1154. 28240603 10.1172/JCI88887 PMC5373853

[24] Agic A , Xu H , Altgassen C , et al. Relative expression of 1, 25‐dihydroxyvitamin D3 receptor, vitamin D 1α‐hydroxylase, vitamin D 24‐hydroxylase, and vitamin D 25‐hydroxylase in endometriosis and gynecologic cancers. Reprod Sci. 2007;14(5):486‐497. 17913968 10.1177/1933719107304565

[25] Moukayed M , Grant WB . The roles of UVB and vitamin D in reducing risk of cancer incidence and mortality: a review of the epidemiology, clinical trials, and mechanisms. Rev Endocr Metab Disord. 2017;18(2):167‐182. 28213657 10.1007/s11154-017-9415-2

[26] Dovnik A , Dovnik NF . Vitamin D and ovarian cancer: systematic review of the literature with a focus on molecular mechanisms. Cell. 2020;9(2):335. 10.3390/cells9020335 PMC7072673 32024052

[27] Abbas MA , Taha MO , Disi AM , Shomaf M . Regression of endometrial implants treated with vitamin D3 in a rat model of endometriosis. Eur J Pharmacol. 2013;715(1–3):72‐75. 23810684 10.1016/j.ejphar.2013.06.016

[28] Yildirim B , Guler T , Akbulut M , Oztekin O , Sariiz G . 1–alpha, 25–dihydroxyvitamin D3 regresses endometriotic implants in rats by inhibiting neovascularization and altering regulation of matrix metalloproteinase. Postgrad Med. 2014;126(1):104‐110. 24393757 10.3810/pgm.2014.01.2730

[29] Delbandi A‐A , Mahmoudi M , Shervin A , Zarnani A‐H . 1,25‐dihydroxy vitamin D3 modulates endometriosis‐related features of human endometriotic stromal cells. Am J Reprod Immunol. 2016;75(4):461‐473. 26691009 10.1111/aji.12463

[30] Miyashita M , Koga K , Izumi G , et al. Effects of 1,25‐dihydroxy vitamin D3 on endometriosis. J Clin Endocrinol Metab. 2016;101(6):2371‐2379. 27035829 10.1210/jc.2016-1515

[31] American Fertility Society, Revised American . Fertility Society classification of endometriosis. Fertil Steril. 1985;43:351‐352. 3979573 10.1016/s0015-0282(16)48430-x

[32] Kolahdouz‐Mohammadi R , Shidfar F , Khodaverdi S , et al. Resveratrol treatment reduces expression of MCP‐1, IL‐6, IL‐8 and RANTES in endometriotic stromal cells. J Cell Mol Med. 2021;25(2):1116‐1127. 33325132 10.1111/jcmm.16178 PMC7812293

[33] Arablou T , Delbandi A‐A , Khodaverdi S , et al. Resveratrol reduces the expression of insulin‐like growth factor‐1 and hepatocyte growth factor in stromal cells of women with endometriosis compared with nonendometriotic women. Phytother Res. 2019;33(4):1044‐1054. 30838714 10.1002/ptr.6298

[34] Hoe E , Nathanielsz J , Toh ZQ , et al. Anti‐inflammatory effects of vitamin D on human immune cells in the context of bacterial infection. Nutrients. 2016;8(12):806. 10.3390/nu8120806 PMC5188461 27973447

[35] Hollis BW . Assessment of vitamin D status and definition of a normal circulating range of 25‐hydroxyvitamin D. Curr Opin Endocrinol Diabetes Obes. 2008;15(6):489‐494. 18971676 10.1097/MED.0b013e328317ca6c

[36] Rashidi N , Mirahmadian M , Jeddi‐Tehrani M , et al. Lipopolysaccharide‐ and lipoteichoic acid‐mediated pro‐inflammatory cytokine production and modulation of TLR2, TLR4 and MyD88 expression in human endometrial cells. J Reprod Infertil. 2015;16(2):72‐81. 25927023 PMC4386089

[37] Islam S , Hassan F , Mu MM , et al. Piceatannol prevents lipopolysaccharide (LPS)‐induced nitric oxide (NO) production and nuclear factor (NF)‐κB activation by inhibiting IκB kinase (IKK). Microbiol Immunol. 2004;48(10):729‐736. 15502405 10.1111/j.1348-0421.2004.tb03598.x

[38] Gmyrek GB , Sozanski R , Jerzak M , et al. Evaluation of monocyte chemotactic protein‐1 levels in peripheral blood of infertile women with endometriosis. Eur J Obstet Gynecol Reprod Biol. 2005;122(2):199‐205. 15893866 10.1016/j.ejogrb.2005.03.019

[39] Zong LL , Li YL , Ha XQ . Determination of HGF concentration in serum and peritoneal fluid in women with endometriosis. Di Yi Jun Yi Da Xue Xue Bao. 2003;23(8):757‐760. 12919890

[40] Garcia‐Velasco JA , Seli E , Arici A . Regulation of monocyte chemotactic protein‐1 expression in human endometrial stromal cells by integrin‐dependent cell adhesion. Biol Reprod. 1999;61(2):548‐552. 10411539 10.1095/biolreprod61.2.548

[41] Yoshimura T , Leonard EJ . Secretion by human fibroblasts of monocyte chemoattractant protein‐1, the product of gene JE. J Immunol. 1990;144(6):2377‐2383. 2313097

[42] Arici A , MacDonald PC , Casey ML . Regulation of monocyte chemotactic protein‐1 gene expression in human endometrial cells in cultures. Mol Cell Endocrinol. 1995;107(2):189‐197. 7768330 10.1016/0303-7207(94)03442-v

[43] Wang YC , Hsieh CC , Kuo HF , et al. Effect of vitamin D3 on monocyte chemoattractant protein 1 production in monocytes and macrophages. Acta Cardiol Sin. 2014;30(2):144‐150. 27122781 PMC4805020

[44] Jain SK , Micinski D . Vitamin D upregulates glutamate cysteine ligase and glutathione reductase, and GSH formation, and decreases ROS and MCP‐1 and IL‐8 secretion in high‐glucose exposed U937 monocytes. Biochem Biophys Res Commun. 2013;437(1):7‐11. 23770363 10.1016/j.bbrc.2013.06.004 PMC4063448

[45] Gao D , Trayhurn P , Bing C . 1,25‐Dihydroxyvitamin D3 inhibits the cytokine‐induced secretion of MCP‐1 and reduces monocyte recruitment by human preadipocytes. Int J Obes (Lond). 2013;37(3):357‐365. 22508334 10.1038/ijo.2012.53 PMC3428854

[46] Yoshida S , Harada T , Mitsunari M , et al. Hepatocyte growth factor/Met system promotes endometrial and endometriotic stromal cell invasion via autocrine and paracrine pathways. J Clin Endocrinol Metab. 2004;89(2):823‐832. 14764801 10.1210/jc.2003-030874

[47] Yerlikaya G , Balendran S , Pröstling K , et al. Comprehensive study of angiogenic factors in women with endometriosis compared to women without endometriosis. Eur J Obstet Gynecol Reprod Biol. 2016;204:88‐98. 27541444 10.1016/j.ejogrb.2016.07.500

[48] Khan KN , Masuzaki H , Fujishita A , Kitajima M , Sekine I , Ishimaru T . Immunoexpression of hepatocyte growth factor and c‐Met receptor in the eutopic endometrium predicts the activity of ectopic endometrium. Fertil Steril. 2003;79(1):173‐181. 12524084 10.1016/s0015-0282(02)04535-1

[49] Delbandi A‐A , Mahmoudi M , Shervin A , Heidari S , Kolahdouz‐Mohammadi R , Zarnani A‐H . Evaluation of apoptosis and angiogenesis in ectopic and eutopic stromal cells of patients with endometriosis compared to non‐endometriotic controls. BMC Womens Health. 2020;20(1):3. 31906916 10.1186/s12905-019-0865-4 PMC6945780

[50] Sugawara J , Fukaya T , Murakami T , Yoshida H , Yajima A . Increased secretion of hepatocyte growth factor by eutopic endometrial stromal cells in women with endometriosis. Fertil Steril. 1997;68(3):468‐472. 9314916 10.1016/s0015-0282(97)00226-4

[51] Zhang X , Nie D , Zhang L , Liu X . Study on diagnostic values and pathological conditions of serum HGF and CA199 in endometriosis. Am J Transl Res. 2021;13(4):2849‐2857. 34017448 PMC8129284

[52] Inaba M , Koyama H , Hino M , et al. Regulation of release of hepatocyte growth factor from human promyelocytic leukemia cells, HL‐60, by 1,25‐dihydroxyvitamin D3, 12‐O‐tetradecanoylphorbol 13‐acetate, and dibutyryl cyclic adenosine monophosphate. Blood. 1993;82(1):53‐59. 8391878

[53] Chattopadhyay N , MacLeod RJ , Tfelt‐Hansen J , Brown EM . 1α, 25 (OH) 2‐vitamin D3 inhibits HGF synthesis and secretion from MG‐63 human osteosarcoma cells. Am J Physiol Endocrinol Metab. 2003;284(1):E219‐E227. 12388161 10.1152/ajpendo.00247.2002

[54] Mu F , Hankinson SE , Schernhammer E , Pollak MN , Missmer SA . A prospective study of insulin‐like growth factor 1, its binding protein 3, and risk of endometriosis. Am J Epidemiol. 2015;182(2):148‐156. 26121987 10.1093/aje/kwv037 PMC4493981

[55] Milingos D , Katopodis H , Milingos S , et al. Insulin‐like growth factor–1 isoform mRNA expression in women with endometriosis: eutopic endometrium versus endometriotic cyst. Ann N Y Acad Sci. 2006;1092:434‐439. 17308170 10.1196/annals.1365.042

[56] Milingos DS , Philippou A , Armakolas A , et al. Insulinlike growth factor‐1Ec (MGF) expression in eutopic and ectopic endometrium: characterization of the MGF E‐peptide actions in vitro. Mol Med. 2011;17(1–2):21‐28. 20844834 10.2119/molmed.2010.00043 PMC3022988

[57] Zhou Y , Zeng C , Li X , et al. IGF‐I stimulates ERβ and aromatase expression via IGF1R/PI3K/AKT‐mediated transcriptional activation in endometriosis. J Mol Med (Berl). 2016;94(8):887‐897. 26899323 10.1007/s00109-016-1396-1

[58] Zhou W‐D , Yang H‐M , Wang Q , et al. SB203580, a p38 mitogen‐activated protein kinase inhibitor, suppresses the development of endometriosis by down‐regulating proinflammatory cytokines and proteolytic factors in a mouse model. Hum Reprod. 2010;25(12):3110‐3116. 20956267 10.1093/humrep/deq287

[59] Xie SP , James SY , Colston KW . Vitamin D derivatives inhibit the mitogenic effects of IGF‐I on MCF‐7 human breast cancer cells. J Endocrinol. 1997;154(3):495‐504. 9379127 10.1677/joe.0.1540495

[60] Xie SP , Pirianov G , Colston KW . Vitamin D analogues suppress IGF‐I signalling and promote apoptosis in breast cancer cells. Eur J Cancer. 1999;35(12):1717‐1723. 10674019 10.1016/s0959-8049(99)00200-2

[61] Pirianov G , Colston KW . Interaction of vitamin D analogs with signaling pathways leading to active cell death in breast cancer cells. Steroids. 2001;66(3–5):309‐318. 11179739 10.1016/s0039-128x(00)00201-4

[62] Ngô C , Chéreau C , Nicco C , Weill B , Chapron C , Batteux F . Reactive oxygen species controls endometriosis progression. Am J Pathol. 2009;175(1):225‐234. 19498006 10.2353/ajpath.2009.080804 PMC2708809

[63] Bedaiwy MA , Falcone T , Sharma RK , et al. Prediction of endometriosis with serum and peritoneal fluid markers: a prospective controlled trial. Hum Reprod. 2002;17(2):426‐431. 11821289 10.1093/humrep/17.2.426

[64] Lousse JC , Van Langendonckt A , González‐Ramos R , Defrère S , Renkin E , Donnez J . Increased activation of nuclear factor‐kappa B (NF‐κB) in isolated peritoneal macrophages of patients with endometriosis. Fertil Steril. 2008;90(1):217‐220. 17889859 10.1016/j.fertnstert.2007.06.015

[65] González‐Ramos R , Donnez J , Defrère S , et al. Nuclear factor‐kappa B is constitutively activated in peritoneal endometriosis. Mol Hum Reprod. 2007;13(7):503‐509. 17483545 10.1093/molehr/gam033

[66] Kaponis A , Iwabe T , Taniguchi F , et al. The role of NF‐kappaB in endometriosis. Front Biosci. 2012;4:1213‐1234. 10.2741/s327 22652867

[67] Stio M , Martinesi M , Bruni S , et al. The Vitamin D analogue TX 527 blocks NF‐κB activation in peripheral blood mononuclear cells of patients with Crohn's disease. J Steroid Biochem Mol Biol. 2007;103(1):51‐60. 17049230 10.1016/j.jsbmb.2006.07.008

[68] Cohen‐Lahav M , Shany S , Tobvin D , Chaimovitz C , Douvdevani A . Vitamin D decreases NFκB activity by increasing IκBα levels. Nephrol Dial Transplant. 2006;21(4):889‐897. 16455676 10.1093/ndt/gfi254

[69] Zhang Y , Leung DYM , Richers BN , et al. Vitamin D inhibits monocyte/macrophage proinflammatory cytokine production by targeting MAPK phosphatase‐1. J Immunol. 2012;188(5):2127‐2135. 22301548 10.4049/jimmunol.1102412 PMC3368346

[70] Wu F‐S , Zheng S‐S , Wu L‐J , et al. Calcitriol inhibits the growth of MHCC97 heptocellular cell lines by down‐modulating c‐met and ERK expressions. Liver Int. 2007;27(5):700‐707. 17498257 10.1111/j.1478-3231.2007.01487.x

[71] Lundqvist J , Hansen SK , Lykkesfeldt AE . Vitamin D analog EB1089 inhibits aromatase expression by dissociation of comodulator WSTF from the CYP19A1 promoter—a new regulatory pathway for aromatase. Biochim Biophys Acta. 2013;1833(1):40‐47. 23085504 10.1016/j.bbamcr.2012.10.012

[72] Hustmyer FG , Girasole G , Manolagas SC . Signal‐dependent pleiotropic regulation of lymphocyte proliferation and cytokine production by 1,25‐dihydroxyvitamin D3: potent modulation of the hormonal effects by phorbol esters. Immunology. 1992;77(4):520‐526. 1493924 PMC1421646

[73] Saggese G , Federico G , Balestri M , Toniolo A . Calcitriol inhibits the PHA‐induced production of IL‐2 and IFN‐gamma and the proliferation of human peripheral blood leukocytes while enhancing the surface expression of HLA class II molecules. J Endocrinol Invest. 1989;12(5):329‐335. 2504804 10.1007/BF03349999

[74] Burney RO , Giudice LC . Pathogenesis and pathophysiology of endometriosis. Fertil Steril. 2012;98(3):511‐519. 22819144 10.1016/j.fertnstert.2012.06.029 PMC3836682

